# Pr^3+^-Activated Sr_2_LaF_7_ Nanoparticles as a Single-Phase White-Light-Emitting Nanophosphor

**DOI:** 10.3390/nano15100717

**Published:** 2025-05-09

**Authors:** Bojana Milićević, Aleksandar Ćirić, Katarina Milenković, Zoran Ristić, Jovana Periša, Željka Antić, Miroslav D. Dramićanin

**Affiliations:** Centre of Excellence for Photoconversion, Vinča Institute of Nuclear Sciences—National Institute of the Republic of Serbia, University of Belgrade, 11000 Belgrade, Serbia; aleksandar.ciric@ff.bg.ac.rs (A.Ć.); katarina.milenkovic@vinca.rs (K.M.); risticz@vin.bg.ac.rs (Z.R.); zeljka.antic@vinca.rs (Ž.A.)

**Keywords:** nanophosphor, fluorides, photoluminescence, Pr^3+^ emission, light-based applications

## Abstract

Sr_2_LaF_7_:*x*Pr^3+^ (*x* = 0.2, 1, 2, 3, 5, 10, and 25 mol%) nanophosphors with a cubic Fm3m structure were hydrothermally synthesized, forming nearly spherical nanoparticles with an average diameter of approximately 32 nm. Diffuse reflectance measurement and excitation spectra showed a primary excitation peak of Pr^3+^ at 443 nm, corresponding to the ground state to the ^3^P_2_ level transition. Upon blue light excitation, Pr^3+^-activated Sr_2_LaF_7_ nanophosphors showed rich emission structure across the visible region of the spectrum, with blue (~483 nm), green (~525 nm), orange (~600 nm), and red (~640 nm) emissions, blue and orange being the most prominent ones. The relative intensities of these emissions varied with Pr^3+^ concentration, leading to tunable emission colors. The chromaticity showed slight variation with the Pr^3+^ content (0.350 < x < 0.417, 0.374 < y < 0.380), while the CCT value increased from 3118 K to 4901 K as the doping concentration increased. The optimized Sr_2_LaF_7_ with 2 mol% Pr^3+^ had the most intense emission with correlated color temperature (CCT) of 3628 K, corresponding to the warm white color. The proposed Pr^3+^-doping strategy offers valuable insights into discovering or optimizing single-phase phosphors for white-light-emitting applications.

## 1. Introduction

In recent decades, researchers have been striving to solve the challenge of achieving stable, high-quality white light in LED devices. Two primary approaches have dominated so far. The first approach combines InGaN blue LED chips with YAG:Ce phosphors; however, insufficient red emission results in a low color rendering index (CRI) and unstable light color owing to sensitivity to processing conditions. The second method utilizes UV or near-UV chips to excite distinct red, green, and blue phosphors, enabling wider spectral output, adjustable CRI, and desirable CCT values. However, this method poses challenges, particularly in the controlled synthesis of these phosphors with uniform particle sizes to prevent agglomeration, which is a complex and time-consuming process. To address these limitations, there has been growing interest in single-phase white-light phosphors, which emit white light from a single host material and offer benefits such as simplified fabrication, reduced production costs, and improved reproducibility. These phosphors encompass a broad spectrum of material hosts, such as fluorides, aluminates, silicates, phosphates, molybdates, and tungstates [1,2,3,4,5,6].

Preferably, host matrices should (i) exhibit non-hygroscopic behavior to maintain stability in air and aqueous environments, (ii) possess a low phonon frequency to minimize non-radiative relaxation losses and enhance emission efficiency, and (iii) feature a wide band gap to facilitate allowed electronic transitions of the dopant ions while preventing self-absorption [7]. The oxides’ hosts exhibit high phonon frequencies (>500 cm^−1^) and good chemical stability. Halide materials possess low phonon frequencies (≤300 cm^−1^); however, their hygroscopic nature restricts their practical applications. Fluorides stand apart from other hosts by having a phonon frequency in the intermediate range (300–500 cm^−1^), good chemical stability, wide optical transmission range, and anionic conductivity [8].

Notably, luminescence-based materials activated by Pr^3+^ ions have a broad range of emissions across the ultraviolet, visible, and infrared spectral regions, arising from interconfigurational $\left[Xe\right]{4f}^{1}{5d}^{1}\to [Xe]{4f}^{2}\;$and intraconfigurational $\left[Xe\right]{4f}^{2}\to [Xe]{4f}^{2}$ electronic transitions [9,10]. These may occur in any form, such as downconversion, downshifting, scintillation, or upconversion. The energy difference between the $\left[Xe\right]{4f}^{1}{5d}^{1}$ and $\left[Xe\right]{4f}^{2}\;$configurations in free Pr^3+^ ions is approximately 62,000 cm^−1^ [9,11]. However, upon doping into crystals, this energy difference considerably reduces because of the crystal field splitting of the $\left[Xe\right]{4f}^{1}{5d}^{1}$ configuration. The degree of energy difference reduction varies significantly across host matrices, influenced by factors such as spectroscopic ion charge density, polarizability, metal–ligand distances, and site symmetry around Pr^3+^ [11,12].

So far, various Pr^3+^-activated fluoride materials have been investigated, providing valuable insights into their potential applications in lighting, displays, sensing, healthcare, and security. Several studies have shown that Pr^3+^-doped fluorides such as LiYF_4_ [13,14,15,16], LiGdF_4_ [17,18], LiLuF_4_ [19], BaY_2_F_8_ [20], and KY_3_F_10_ [21] are promising candidates for solid-state lasers due to the effective emissions from ^3^P_0_ across the visible and near-infrared spectral ranges. Mono-dispersed Pr^3+^-doped β-NaYF_4_, with an absolute sensitivity of 0.01352 K^−1^ at 300 K, and Pr^3+^-doped YF_3_ exhibiting an absolute sensitivity of 0.012 K^−1^, with a temperature resolution reaching 0.5 K, are promising candidates for temperature sensing [22,23]. Recently, Pr^3+^-doped fluoride materials have gained significant attention for antimicrobial applications [24]. In this context, Yang et al. observed X-ray-activated strong UVC persistent luminescence, linked to the $\left[Xe\right]{4f}^{1}{5d}^{1}\to [Xe]{4f}^{2}$ transition of Pr^3+^, upon releasing trapped electrons in the fluoride elpasolite Cs_2_NaYF_6_ as a host, providing new insights into their potential applications for sterilization, disinfection, and more [25]. Furthermore, materials that can convert visible light into UVC, such as KCaF_3_:Pr^3+^, CsCaF_3_:Pr^3+^, RbCaF_3_:Pr^3+^, and BaYF_5_:Pr^3+^, show strong potential for use in phototherapy, antimicrobial treatments, and photocatalytic processes [26,27]. In addition, β-NaYF_4_:Pr^3+^, a single-phase phosphor, is an excellent candidate for white-light-emitting diodes, demonstrating excellent color stability across temperature variations and a CCT value of 5951 K [28].

The growing interest in nanomaterials has increased the demand for novel compounds with intense emissions and efficient synthesis techniques. Nano-sized, lanthanide-doped mixed metal fluorides, M_2_LnF_7_ (where M = Ca, Sr, Ba and Ln^3+^ = Y, La, Gd, Lu), remain relatively unexplored as phosphors, with only a few studies focusing on M_2_LaF_7_ upconversion nanoparticles [29,30,31]. In this study, cubic Pr^3+^-activated Sr_2_LaF_7_ (Pr^3+^ content: 0.2, 1, 2, 3, 5, 10, and 25 mol%) was synthesized hydrothermally at 180 °C for 20 h. The main excitation peak of Pr^3+^ occurs at 443 nm, and the strongest emission is achieved with an optimal doping concentration of 2 mol%. In addition, the lifetime–concentration relationships show that increasing Pr^3+^ concentration shortens lifetime through enhanced non-radiative pathways. Chromaticity (*x*, *y*) varies slightly, while CCT increases with Pr^3+^-doping. The luminescent properties of Pr^3+^-activated Sr_2_LaF_7_ nanophosphors have not yet been reported in the literature.

## 2. Materials and Methods

### 2.1. Chemicals

Strontium nitrate (Sr(NO_3_)_2_, Haverhill, MA, USA, 99%), lanthanum (III) nitrate hexahydrate (La(NO_3_)_3_ꞏ6H_2_O, Alfa Aesar, Haverhill, MA, USA, 99.99%), praseodymium oxide (Pr_6_O_11_, Haverhill, MA, USA, 99.9%), disodium ethylenediaminetetraacetate dihydrate (EDTA-2Na, C_10_H_14_N_2_O_8_Na_2_ꞏ2H_2_O, Kemika, Zagreb, Croatia, 99%), ammonium fluoride (NH_4_F, Alfa Aesar, Haverhill, MA, USA, 98%), 25% ammonium solution (NH_4_OH, Fisher, Loughborough, Leicestershire, UK), nitric acid (65% HNO_3_, Macron fine chemicals, Center Valley, PA, USA), and deionized water were used as starting materials without additional purification.

### 2.2. Synthesis of SLF:Pr

**Figure 1:**
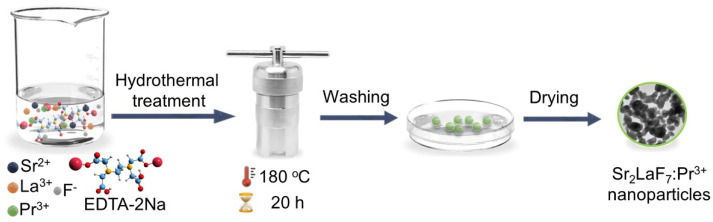
Illustration of the synthesis procedure of SLF:Pr nanophosphors via an EDTA-assisted hydrothermal method.

The total of seven samples of Pr-doped Sr_2_LaF_7_—SLF (Sr_2_La_1−x_Pr*_x_*F_7_, *x* = 0.002, 0.01, 0.02, 0.03, 0.05, 0.10, and 0.25) were synthesized hydrothermally using Sr^2+^ and La^3+^-nitrates, Pr^3+/4+^-oxide, and NH_4_F as precursors and EDTA-2Na as a stabilizer (Figure 1). Typically, to produce 0.89 g of the representative sample—Sr_2_LaF_7_ doped with 2 mol% Pr^3+^—firstly, a stoichiometric quantity of Pr^3+^/^4+^-oxide was dissolved in 3 mL of concentrated nitric acid and evaporated until oxide transformed into nitrate at 120 °C. After that, the nitrates were weighed corresponding to the stoichiometric ratio (specifically, 0.8465 g Sr^2+^-nitrate, 0.8487 g La^3+^-nitrate) and dissolved in deionized water (12.5 mL) containing Pr-nitrates under continuous stirring at room temperature for 30 min. The resulting solution was then combined with a transparent solution containing 0.7445 g EDTA-2Na in 12.5 mL in water (molar ratio EDTA-2Na:La = 1:1). Subsequently, a 10 mL aqueous solution containing 0.8889 g of NH_4_F (molar ratio NH_4_F:La = 12:1) was introduced and stirred vigorously for 1 h, resulting in the formation of a white complex. The mixture’s pH was modified to about 6 by adding 800 μL of NH_4_OH. After being sealed in a 100 mL Teflon-lined autoclave, the solution was heated at 180 °C for 20 h. Upon natural cooling, the products were centrifuged, washed twice with water and once with a 1:1 ethanol/water mixture to remove any remaining residues, and then dried in an air atmosphere at 80 °C for 4 h. SLF phosphors with different Pr^3+^ concentrations (x = 0.2, 1, 2, 3, 5, 10, and 25 mol%) relative to La^3+^ ions were synthesized following the procedure outlined above. Table 1 shows the specific precursor quantities used to prepare 0.002 mol of Sr_2_La_1−x_Pr*_x_*F_7_ (*x* = 0.002, 0.01, 0.02, 0.03, 0.05, 0.10, and 0.25) samples.

### 2.3. Characterization

X-ray diffraction (XRD) analysis was conducted using a Rigaku SmartLab system (Tokyo, Japan), employing Cu Kα radiation (30 mA, 40 kV) over a 2θ range of 10° to 90°. The diffraction data were collected with a step size of 0.02° and a scanning rate of 1°/min across the analyzed 2θ range. The microstructure of the samples was examined using a Tecnai GF20 transmission electron microscope (TEM) operating at 200 kV (Hillsboro, OR, USA). ImageJ software (Open-source software, https://imagej.net/, accessed on 6 May 2025) was used to determine the average particle size. Diffuse reflectance spectra were obtained using a Shimadzu UV-2600 (Shimadzu Corporation, Tokyo, Japan) spectrophotometer equipped with an ISR-2600 integrated sphere, with BaSO_4_ serving as the reference standard. Luminescence measurements were carried out using a 450 nm laser as the excitation source. Emission and excitation spectra were recorded with a Fluorolog-3 Model FL3-221 spectrofluorometer system (Horiba-Jobin-Yvon, Longjumeau, France), utilizing a 450 nm laser for excitation. Excited-state lifetime evaluations were performed using an FHR1000 high-resolution monochromator (Horiba Jobin Yvon), an ICCD camera (Horiba Jobin Yvon 3771, Longjumeau, France), and a 450 nm laser source. Quantum efficiency measurements were performed using a home-built system, consisting of Ocean Insight IDP-REF 38.1 mm integrating sphere fiber coupled to the 450 nm laser light source on the reference port and the OCEAN-FX-XR1-ES extended range spectrometer (Winter Park, FL, USA) on the sample port of the sphere, using BaSO_4_ as the standard reference. All data were collected at room temperature.

## 3. Results and Discussion

### 3.1. Structure and Morphology

**Figure 2:**
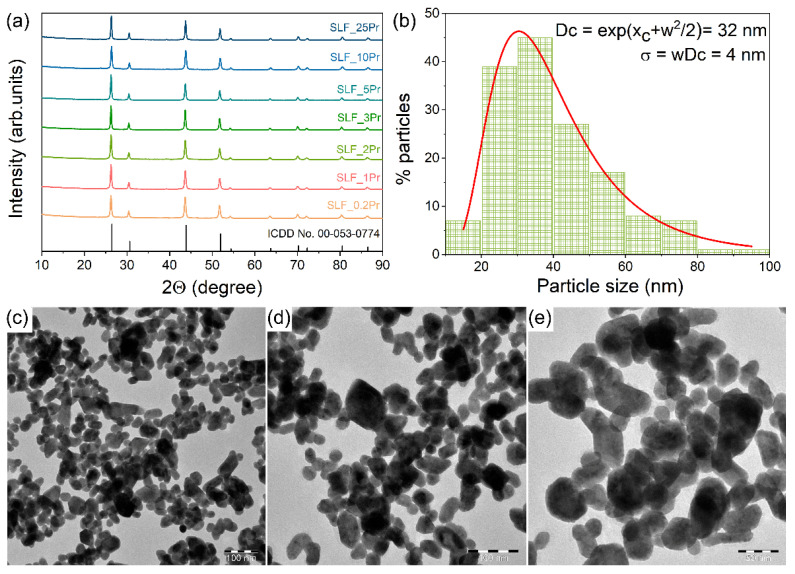
(**a**) XRD patterns of hydrothermally synthesized Sr_2_LaF_7_:*x*Pr (*x* = 0.2, 1, 2, 3, 5, 10, and 25 mol%) samples presented with the ICDD card No. 00–053–0774 for pure Sr_2_LaF_7_. (**b**) Particle size distribution histogram. (**c**–**e**) TEM images of the representative SLF:2Pr sample with different magnification ×40,000, ×60,000, and ×100,000.

The M_x_LnF_2x+3_ fluorides crystallize in a cubic structure with the Fm3m space group [30]. XRD patterns of Sr_2_La_1−*x*_Pr*_x_*F_7_ (*x* = 0.002, 0.010, 0.020, 0.030, 0.050, 0.100, and 0.250) nanophosphors with varying Pr^3+^ concentrations are shown in Figure 2a. Although Pr^3+^ ions were introduced, the main diffraction peaks appearing at approximately 2θ = 26.3, 30.4, 43.7, 51.7, 54.2, 63.5, 70.0, 72.1, and 80.3° remained unchanged. These peaks correspond to the 111, 200, 220, 311, 222, 400, 331, 420, and 422 crystal planes, respectively, and show excellent agreement with the standard diffraction data for pure Sr_2_LaF_7_, as documented in ICDD card No. 00–053–0774. The patterns revealed no evidence of additional phases or impurity-related diffraction peaks. The sharp diffraction peaks indicate a good crystallinity of SLF nanophosphors. Table 2 shows the results of the structural analysis: crystallite size (*CS*), microstrain values, unit cell parameters (*a*), unit cell volume (*CV*), and the parameters of the data fitting (R_wp_, R_p_, R_e_, GOF) of SLF:*x*Pr (*x* = 0.2, 1, 2, 3, 5, 10, and 25 mol%) nanophosphors.

The SLF:Pr nanophosphors were synthesized using an EDTA-assisted hydrothermal method, where EDTA, an effective complexing agent, enhances the dispersibility of crystalline seeds by producing [Sr-EDTA]^2+^ and [La-EDTA]^+^ complexes, thus inhibiting the aggregation of SLF particles during the hydrothermal treatment. The resulting morphology and particle size distribution of the representative SLF:2Pr sample, observed at different magnifications, are presented in the TEM images and histogram shown in Figure 2b–e. The nanoparticles exhibit an almost spherical morphology, with an average particle size of approximately 32 ± 4 nm, as estimated from a histogram based on roughly 200 particles and fitted with a log-normal distribution (Figure 2b). High-particle-density zones, which form aggregates and areas with lower dispersed particles, are noticed at lower magnification (Figure 2c), whereas the variation in particle size and shape can be visualized at higher magnification in Figure 2e.

### 3.2. Photoluminescence Properties of SLF:Pr

Pr^3+^ ions provide emissions originating from 4f5d→4f^2^ and 4f^2^→4f^2^ electronic transitions that cover the UVC to NIR spectral range. If the lowest-energy 4f5d level has lower energy than the 4f^2 1^S_0_ state, the parity-allowed transitions from 4f5d exhibit UV emission as a dominant one. In the case of fluorides, 4f5d levels usually have higher energy than the ^1^S_0_ state, which facilitates VIS-NIR emissions from a cascade 4f^2^ → 4f^2^ transitions. If 4f5d and ^1^S_0_ are close in energy, both types of transitions occur.

**Figure 3:**
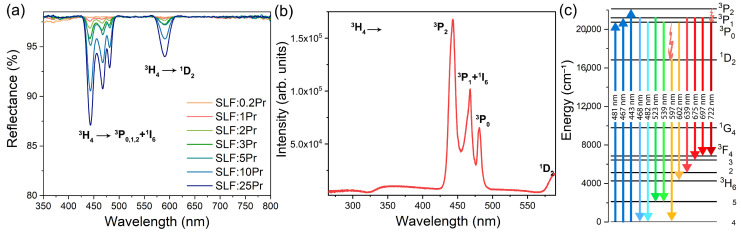
(**a**) Diffuse reflectance spectra of SLF:*x*Pr (*x* = 0.2, 1, 2, 3, 5, 10, and 25 mol%) samples. (**b**) The excitation spectrum of SLF:2Pr obtained by monitoring λ_em_ = 600 nm. (**c**) Energy level diagram of the mechanisms of Pr^3+^ emission.

Figure 3a displays the diffuse reflection spectra of Sr_2_LaF_7_:*x*Pr (*x* = 0.2, 1, 2, 3, 5, 10, and 25 mol%) nanophosphors recorded at room temperature across the visible wavelength range. Pr^3+^ absorptions from ^3^H_4_ → ^3^P_0,1,2_+^1^I_6_ and ^3^H_4_ → ^1^D_2_ transitions are evident in the blue and orange-red spectral areas, with the most intense absorption peak at 443 nm attributed to the ^3^H_4_ → ^3^P_2_ electronic transition, which is known for its relatively high absorption cross-section [32].

**Figure 4:**
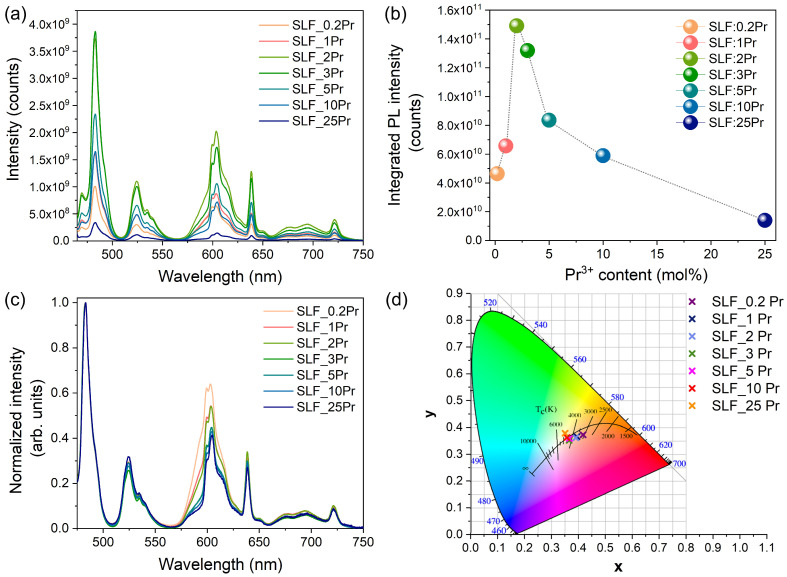
(**a**) Emission spectra after 450 nm laser irradiation and (**b**) integrated emission intensity as a function of Pr^3+^ concentration. (**c**) Normalized emission spectra. (**d**) CIE chromaticity diagram of SLF:*x*Pr (*x* = 0.2, 1, 2, 3, 5, 10, and 25 mol%) samples obtained from emission spectra in (**a**). The solid black line represents the Planckian locus, with several color temperatures indicated along it [41].

Figure 3b shows the excitation spectrum recorded with a 600 nm emission wavelength, exhibiting a spectral profile similar to Figure 3a. The primary excitation peak originates from the ground state to the ^3^P_2_ level of Pr^3+^ at 443 nm. The ^3^P_1_ and ^1^I_6_ levels overlap at 467 nm, while the ^3^P_0_ level appears as the sharpest peak at 481 nm due to its J = 0 total angular momentum, which prevents Stark splitting. The excitation into the ^1^D_2_ level is observed at 590 nm. These observations indicate that the ^3^P_2_, ^3^P_1_+^1^I_6_, ^3^P_0_, and ^1^D_2_ levels are positioned at energies of 22,573 cm^−1^, 21,413 cm^−1^, 20,790 cm^−1^, and 16,949 cm^−1^, respectively (see Figure 3c). The excitation spectrum aligns well with the 450 nm laser light used to generate the emissions recorded in Figure 4a. From the ^3^P_2_ level, the electrons non-radiatively de-excite to the lowest level of the ^3^P_J_ multiplet by the multiphonon mechanism [33].

The emission spectra feature transitions including ^3^P_1_ → ^3^H_4_ at 468 nm, ^3^P_1_ → ^3^H_4_ at 483 nm, ^3^P_1_ → ^3^H₅ at 525 nm, ^3^P_0_ → ^3^H₅ at 539 nm, ^1^D_2_ → ^3^H_4_ at 597 nm, ^3^P_0_ → ^3^H_6_ at 602 nm, ^3^P_1_ → ^3^F_2_ at 640 nm, ^3^P_1_ → ^3^F_3_ at 675 nm, ^3^P_1_ → ^3^F_4_ at 697 nm, and ^3^P_0_ → ^3^F_4_ at 722 nm, similar to what was reported for other Pr^3+^-doped materials [28,34,35,36,37,38]. Here, the observed emission peaks are broader, which can be attributed to the nanoscale dimensions of the analyzed SLF:*x*Pr (*x* = 0.2, 1, 2, 3, 5, 10, and 25 mol%) particles. The emissions from the ^3^P_1_ level occur because this level is readily populated at room temperature, owing to its small energy separation from the ^3^P_0_ level [22]. According to the relation for the fractional thermal population [39], the percentage of optical centers in ^3^P_1_+^1^I_6_ levels is about 30% at 300 K. The ^3^P_2_ level lying at about 1750 cm^−1^ from the ^3^P_0_ level is energetically too far to be significantly populated at room temperature.

The integrated emission intensity (Figure 4b) reveals that 2 mol% Pr^3+^-doping yields the strongest emission, representing the optimal concentration after which concentration quenching begins. Thus, 2 mol% is the optimal doping concentration for the Sr_2_LaF_7_ host by Pr^3+^ ions.

The normalized spectra presented in Figure 4c show that as concentration increases, the peak at ~600 nm diminishes relative to the blue emission. This peak comprises overlapping ^1^D_2_ and ^3^P_0_ emissions, and since the spectra are already normalized to the ^3^P_0_ emissions, this means that there is a decrease in ^1^D_2_ emissions with increasing concentration. This changing emission ratio between ^1^D_2_ and ^3^P_0_ levels stems from non-radiative processes. Higher Pr^3+^ concentrations reduce inter-ionic distances, enhancing cross-relaxation mechanisms that preferentially quench the ^1^D_2_ level while affecting ^3^P_0_ emission less [40]. This selective quenching explains the increasing I(^3^P_0_)/I(^1^D_2_) ratio at higher concentrations. This reduction in the red component by suppressing the ^1^D_2_ emission is a pathway for color tunability of this phosphor. For the SLF:Pr^3+^ samples, quantum efficiencies were determined to be 16%, 25%, 55%, 51%, 31%, 20%, and 7% for Pr^3+^-dopant concentrations of 0.2%, 1%, 2%, 3%, 5%, 10%, and 25%, respectively.

So far, researchers have typically aimed to design phosphors whose chromaticity values align with the blackbody radiator curve, also known as the Planckian locus. When this is achieved, the light source should match the color temperature of a blackbody at the same physical temperature. However, the concept can still be applied even when a phosphor’s chromaticity deviates slightly from the blackbody curve. In such cases, the correlated color temperature (CCT) is used, which represents the temperature of the blackbody radiation that has chromaticity nearly matching that of the source. CCT is a key metric influencing how warm or cool the phosphors’ emission appears to human perception. For example, incandescent bulbs, common in homes, emit warm light and typically have CCT values between 2700 and 3000 K [42]. On the other hand, fluorescent lights, which appear cool/daylight white, usually fall within the 4000 to 6000 K range due to the lack of red emission [42]. Figure 4d shows that the chromaticity coordinates of the SLF:*x*Pr (*x* = 0.2, 1, 2, 3, 5, 10, and 25 mol%) phosphors are located near the Planckian locus and exhibit minor changes with increasing Pr^3+^-doping concentration. Also, the CCT values given in Table 3 are higher with an increase in Pr^3+^-doping concentration. This is consistent with the increase in the I(^3^P_0_)/I(^1^D_2_) emission intensity ratio. Previous studies on single-phase β-NaYF_4_:Pr^3+^ and KYF_4_:Pr^3+^ reported the chromaticity coordinates of cool white light with CCT values between 4500 and 6800 K due to the lack of red emission [28,39]. Here, CCT values change with Pr^3+^-doping concentration and range from 3100 to 4900 K. Due to strong red emission, the optimized SLF:2Pr sample emits warm white light at (0.390, 0.363) and a CCT of 3628 K. Our findings are similar to BaY_2_F_8_:Pr^3+^ and KY_3_F_10_:Pr^3+^ phosphors, as given in Table 3 showing previously reported chromaticity coordinates of white light with CCT values for single-phase white-light-emitting fluoride and results obtained in this work. However, chromaticity values (x, y) of optimized SLF:2Pr are much closer to the Planckian locus when compared with BaY_2_F_8_:Pr^3+^ and KY_3_F_10_:Pr^3+^ phosphors, indicating that its emitted light exhibits a color most similar to natural warm white light.

According to its excitation spectrum, SLF:*x*Pr (*x* = 0.2, 1, 2, 3, 5, 10, and 25 mol%) phosphor is an excellent candidate for light-based applications using commercially available, cost-effective, and powerful blue chips, particularly those operating at 470 nm. Knowing that the LED output light consists of a tunable ratio of LED chip light and phosphor emission [43], it is possible to tune LED emission from cool/daylight white (4000–6000 K) to warm white (2700–3000 K) by choosing the Pr^3+^ content in the SLF phosphor.

**Figure 5:**
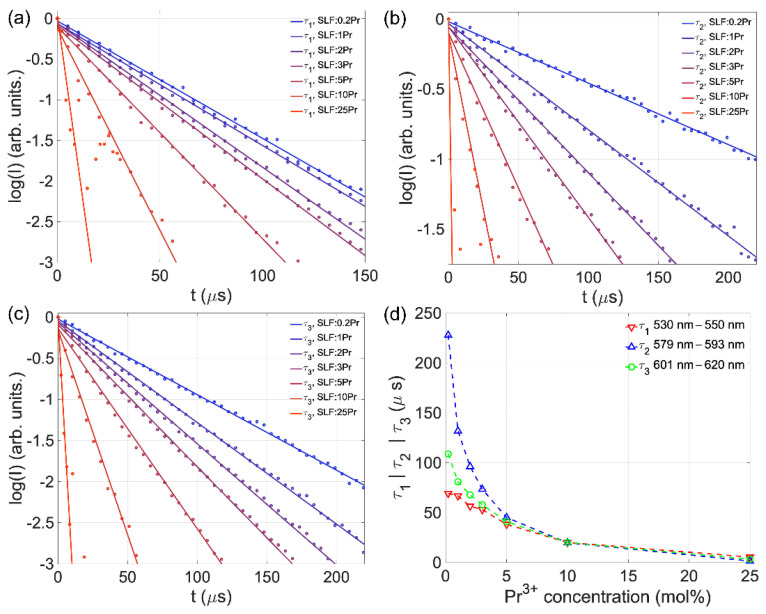
Emission decay curves for (**a**) ^3^P_0_,_1_ levels (τ_1_, 530–550 nm), (**b**) ^1^D_2_ level (τ_2_, 579–593 nm), and (**c**) overlapping ^1^D_2_ and ^3^P_0_ levels (τ_3_, 601–620 nm). (**d**) Lifetime value vs. Pr^3+^ concentration in SLF.

Figure 5a–c displays the temporal dependence of Pr^3+^ luminescence for ^3^P_0_,_1_ levels (τ_1_, 530–550 nm), the ^1^D_2_ level (τ_2_, 579–593 nm), and overlapping ^1^D_2_ and ^3^P_0_ levels (τ_3_, 601–620 nm), with corresponding lifetime–concentration relationships shown in Figure 5d. The thermalized ^3^P_0_,_1_ levels share identical lifetimes. The lifetime values (*τ*) were calculated by fitting the data to normalized intensity decay curves with a single exponential model (Equation (1)):

(1) $$\;I\left(t\right)=I_{0}e^{-\frac{t}{\tau }}$$

where *I*(*t*) represents the corresponding emission intensity at time *t*, *I*_0_ represents the corresponding emission intensity at time *t =* 0 (ideally *I*_0_ *=* 1 for normalized I(t)), and *τ* represents the emission decay constant (excited-state lifetime). Fitting parameters are shown in Table 4 for all the samples in all three wavelength ranges, resulting in goodness-of-fit parameters (R^2^) being above 0.99 for all except for SLF:25Pr, where the lifetime values are approximate and serve a phenomenological role to illustrate the impact of concentration quenching.

The lifetimes of both ^3^P_0_ and ^1^D_2_ levels uniformly decrease with higher Pr^3+^ concentrations, as a result of cross-relaxation effects. At lower Pr^3+^ concentrations, the ^1^D_2_ emission decays more slowly than ^3^P_0_ due to its greater isolation from the next lower level, resulting in less probable multiphonon relaxation. Increasing concentration then shortens all lifetimes through enhanced cross-relaxation non-radiative pathways. The lifetime changes align with the spectral shape variations observed in Figure 5d: ^1^D_2_ lifetime decreases more rapidly with concentration than ^3^P_0_, with both reaching equivalence at 10 mol%. Thus, the lifetime analysis confirms that the cross-relaxation pathways affect the ^1^D_2_ population more than that of the ^3^P_0_ level.

## 4. Conclusions

We have successfully fabricated Pr^3+^-doped Sr_2_LaF_7_ nanophosphors using a hydrothermal approach, achieving a cubic crystal structure and quasi-spherical particle shape of around 32 nm. We have analyzed photoluminescent properties in detail and demonstrated the following:

(i) Sr_2_LaF_7_:Pr nanophosphors exhibit a prominent blue emission at 483 nm, along with additional green, orange, and red emissions.

(ii) The optimal concentration was observed in Sr_2_La_0.08_Pr_0.02_F_7_, which exhibited the strongest emission and a CCT value of 3628 K.

(iii) The analysis of emission lifetimes showed that increasing the Pr^3+^ concentration shortened the lifetimes at all emission wavelengths, particularly for the ^1^D_2_ state, which was more susceptible to concentration-induced quenching than the ^3^P_0_ state.

These findings underscore the potential of SLF:Pr nanophosphors as a single-phase phosphor and their possible use in white LEDs when paired with commercially available, cost-effective blue chips operating at 470 nm. Our future research efforts will be directed toward fabrication and thorough examination of Pr^3+^-activated single-phase microcrystals, with a primary focus on determining their efficiency, temperature stability, and LED fabrication.

## Figures and Tables

**Table 1:** The specific precursor quantities needed for preparing 0.002 mol of Sr_2_La_1−x_Pr*_x_*F_7_ (*x* = 0.002, 0.01, 0.02, 0.03, 0.05, 0.10, and 0.25).

Molecular Formula	Pr^3+^ (mol%)	Abbreviated Name	Precursors (g)				
			Sr(NO_3_)_2_	La(NO_3_)_3_ꞏ6H_2_O	Pr_6_O_11_	NH_4_F	EDTA-2Na
Sr_2_La_0.998_Pr_0.002_F_7_	0.2	SLF:0.2Pr	0.8465	0.8643	0.0007	0.8889	0.7445
Sr_2_La_0.99_Pr_0.01_F_7_	1	SLF:1Pr	0.8465	0.8574	0.0034	0.8889	0.7445
Sr_2_La_0.98_Pr_0.02_F_7_	2	SLF:2Pr	0.8465	0.8487	0.0068	0.8889	0.7445
Sr_2_La_0.97_Pr_0.03_F_7_	3	SLF:3Pr	0.8465	0.8401	0.0102	0.8889	0.7445
Sr_2_La_0.95_Pr_0.05_F_7_	5	SLF:5Pr	0.8465	0.82274	0.0170	0.8889	0.7445
Sr_2_La_0.90_Pr_0.10_F_7_	10	SLF:10Pr	0.8465	0.7794	0.0340	0.8889	0.7445
Sr_2_La_0.75_Pr_0.25_F_7_	25	SLF:25Pr	0.8465	0.6495	0.0851	0.8889	0.7445

**Table 2:** Results of the structural analysis of SLF:*x*Pr (*x* = 0.2, 1, 2, 3, 5, 10, and 25 mol%) nanophosphors.

Pr^3+^ Content (mol%)	0.2	1	2	3	5	10	25
Abbreviated Name	SLF:0.2Pr	SLF:1Pr	SLF:2Pr	SLF:3Pr	SLF:5Pr	SLF:10Pr	SLF:25Pr
*a* = *b* = *c* (Å)	5.8465 (2)	5.8541 (3)	5.8492 (3)	5.84912 (15)	5.8528 (3)	5.83895 (16)	5.85336 (17)
*CV* (Å^3^)	199.84 (3)	200.62 (4)	200.12 (4)	200.11 (2)	200.49 (4)	199.07 (2)	200.55 (2)
*CS* (Å)	185.0 (15)	230.4 (10)	229.9 (2)	268.6 (10)	266.4 (9)	236.80 (8)	293.0 (6)
Strain	0.120 (10)	0.141 (19)	0.146 (4)	0.036 (12)	0.145 (11)	0.1704 (13)	0.124 (6)
GOF	1.4617	1.7782	1.7023	1.6925	2.2436	1.3764	1.3033
* R_wp_	5.37	6.42	6.08	6.26	8.31	5.05	4.90
** R_p_	4.04	4.65	4.42	4.69	5.89	3.71	3.66
*** R_e_	3.67	3.61	3.57	3.70	3.70	3.67	3.76

* R_wp_—the weighted profile factor; ** R_p_—the profile factor; *** R_e_—the expected weighted profile factor; GOF—the goodness of fit.

**Table 3:** Colorimetric parameters of Sr_2_LaF_7_:*x*Pr (*x* = 0.2, 1, 2, 3, 5, 10, and 25 mol%) and previously reported Pr^3+^-activated single-phase white-light-emitting phosphors.

Sample	Pr^3+^ Content (%)	λ_exc_ (nm)	(x, y)	CCT (K)	Reference
β-NaYF_4_:Pr^3+^	0.1	443	(0.354, 0.339)	4563	[28]
	0.5		(0.323, 0.338)	5951	
	1.0		(0.307, 0.335)	6767	
BaY_2_F_8_:Pr^3+^	0.3	457.9	(0.35, 0.32)	4667	[36]
	1.25		(0.38, 0.34)	3724	
	3.0		(0.40, 0.34)	3158	
KYF_4_:Pr^3+^	1.25	457.9	(0.35, 0.31)	4604	[36]
KY_3_F_10_:Pr^3+^	0.3	457.9	(0.37, 0.32)	3861	[36]
Sr_2_LaF_7_:Pr^3+^	0.2	468	(0.417, 0.374)	3105	This work
	1		(0.394, 0.367)	3561	
	2		(0.390, 0.363)	3628	
	3		(0.365, 0.353)	4292	
	5		(0.366, 0.360)	4308	
	10		(0.357, 0.364)	4628	
	25		(0.350, 0.380)	4924	

**Table 4:** Fitting parameters from Equation (1) for 530–550 nm (τ_1_, ^3^P_0_,_1_ levels), 579–593 nm (τ_2_, ^1^D_2_ level), and 601–620 nm (τ_3_, overlapping ^1^D_2_ and ^3^P_0_ levels) ranges.

Sample	Fitting Parameters					
	$I_{0,1}$	$\tau_{1}$ [µs]	$I_{0,2}$	$\tau_{2}$ [µs]	$I_{0,3}$	$\tau_{3}$ [µs]
SLF:0.2Pr	0.969	69.3	0.981	227.9	0.976	108.9
SLF:1Pr	0.938	66.8	0.968	131.9	0.953	81.0
SLF:2Pr	0.940	56.6	0.943	96.2	0.936	67.8
SLF:3Pr	0.919	53.0	0.945	73.4	0.911	57.9
SLF:5Pr	0.904	38.3	0.905	45.1	0.871	41.0
SLF:10Pr	0.887	20.1	0.898	19.9	0.853	20.1
SLF:25Pr	1.035	5.5	0.984	1.5	0.974	3.4

## Data Availability

The original contributions presented in the study are included in the article, further inquiries can be directed to the corresponding author.
